# Comprehensive review of understanding ancient dietary habits using modern analytical techniques

**DOI:** 10.1016/j.fochms.2025.100304

**Published:** 2025-10-10

**Authors:** Nehal S. Ramadan, Magdy M. El-Sayed, Hesham Sameh Ramadan, Mostafa Ismail, Heba Abdelmegeed, Nashwa Gaber, Mahmoud M. Sakr

**Affiliations:** a Chemistry of Tanning Materials and Leather Technology Department, Chemical Industries Research Institute, National Research Centre, Dokki, Cairo 12622, Egypt; b Dairy Science Department, Food Industries Research Institute, National Research Centre, Dokki, Cairo 12622, Egypt; c Department of Pharmaceutical Chemistry, Faculty of Pharmacy, Horus University- Egypt, New Damietta, Egypt; d National Museum of Egyptian Civilization, 17611 Cairo, Egypt; e Chemistry of Natural Compounds Department, Pharmaceutical and Drug Industries Research Institute, National Research Centre, Dokki, Cairo 12622, Egypt; f Permanent Committee and Foreign Missions, Ministry of Antiquities, Giza, Egypt; g Genetic Engineering and Biotechnology Division, National Research Centre, Dokki, Cairo 12622, Egypt

**Keywords:** Ancient diet reconstruction, Biomolecular archaeology, Archaeological remnants, Isotope analysis, Lipid biomarkers, Ancient DNA (aDNA), Proteomics, Metabolomics, Archaeometry

## Abstract

Understanding ancient dietary habits is essential for reconstructing ancient lifestyles. Archaeobotanical and archaeological remains, such as seeds, plant fibers, and pottery, serve as vital indicators of agriculture and daily life. We proposed that contemporary biomolecular and analytical methods provide unique insights into dietary habits through these artifacts. To assess this, we examined recent analytical platforms utilized in isotope analysis, multi-omics techniques viz. genomics, proteomics, lipidomics, and metabolomics applied to food residues, dental calculus, coprolites, and ceramics. Isotope research has shed light on climate changes and human movement; lipid and protein examinations uncovered methods of food preparation and preservation; and multi-omics strategies have enhanced the detail of dietary reconstructions, increasingly aided by artificial intelligence. Ongoing challenges persist, such as sample preservation, contamination, and potential methodological bias. Nevertheless, our review affirms that biomolecular archaeology significantly enhances our understanding of ancient diets and sharpens archaeological interpretations regarding health, subsistence, and interactions between humans and their environment.

## Highlights

• Employed multi-omics methods to unravel ancient dietary habits.
• Showcased isotope analysis that track historical climate changes.
• Identified lipid and protein markers in food residues.
• Evaluated ancient DNA to reconstruct the diets of flora and fauna.
• Highlighted analytical challenges encountered in biomolecular archaeology.

## Graphical abstract

**Figure:**
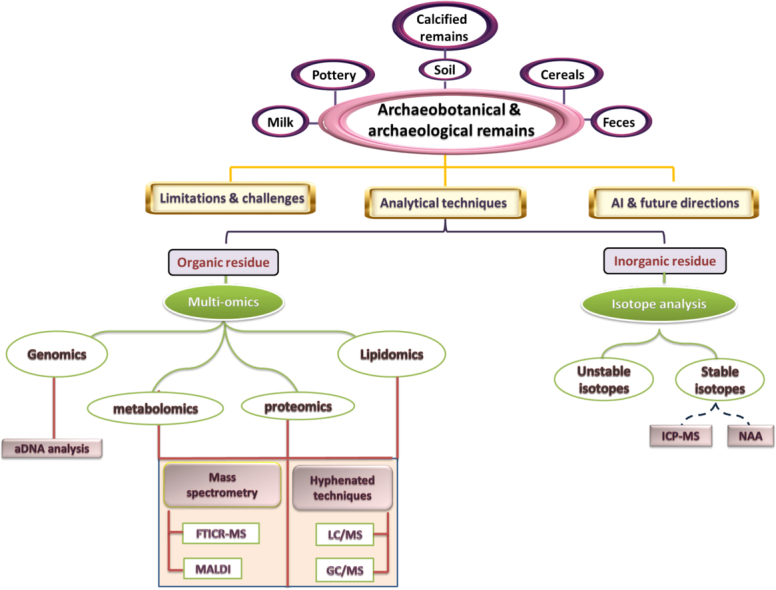

## 1 Introduction

Archaeology is a profoundly interdisciplinary field. Advances in analytical instrumentation and methodologies have expanded archaeological research.

The conceptualization of food as an essential aspect of human existence and as an embodied material culture, underscores its social and cultural significance, making food remnants, cooking, storage, and transport containers as pertinent subjects for archaeological inquiry (Polla & Springer, 2022).

A variety of archaeological dietary food remains, including dairy and non-dairy animal proteins, as well as archaeological plant remains, serve as invaluable repositories of knowledge on the social and environmental conditions prevalent in ancient societies (Cappers & Neef, 2021; Ferrio et al., 2020; Fuller et al., 2023; Lodwick & Rowan, 2022; Siegel & Pearsall, 2023; Vilanova & Porcar, 2020).

In addition to archaeobotanical remains, diverse calcified and mineralized substrates serve as reservoirs for ancient biomolecules. These substrates encompass bones, eggshells, invertebrate shells, coprolites, dental calculus, and keratinous materials, such as hair. Moreover, soil samples obtained from archaeological sites serve as valuable matrices to analyze and identify trapped organic compounds (Grealy et al., 2023; Manzano et al., 2019; Tripp et al., 2022). The initial examination of organic residues was constrained by the lack of suitable methods, relying on melting points and solubility tests (Craig et al., 2020).

Maximizing the informative potential of such remains requires analytical techniques. Biomarkers preserved across archaeological materials, combined with extraction and identification techniques, provide novel insights into past human activities (Polla & Springer, 2022).

The selection of techniques for archaeological research is contingent upon the study's objectives, which may entail establishing the chronological sequence of historical events, identifying biological remnants, or scrutinizing the composition of a specific artifact. Various determinants, such as the material type under investigation, the methodological requisites (e.g., invasive versus non-invasive), and the interpretive potential of the data influence the experimental design and the methodology chosen for archaeological research (Manzano et al., 2019; Vilanova & Porcar, 2020; Vodanović et al., 2023).

A diverse range of techniques, spanning spectroscopic, spectrometric, isotopic, molecular, and genetic methods, has been assimilated from various disciplines and applied to archaeological study. Because archaeological samples are scarce, methods aim to maximize data from minimal material (Badillo-Sanchez, Ruber, Davies-Barrett, Jones, & Inskip, 2023; Boggi et al., 2023). For instance, in modern radiocarbon dating, only about 1 % or a few milligrams are needed instead of several grams, hence keeping integrity after the shift from decay counting to isotope ratio mass spectrometry IR-MS (Polla & Springer, 2022).

Biomolecular archaeology entails the analysis of ancient elements viz. stable isotopes as well as ancient molecules, particularly nucleic acids, proteins, lipids, and carbohydrates many of which are chemically stable and poorly soluble in water, enabling resolution of long-standing questions (Tomei et al., 2023; Vallejo et al., 2022). This field is applied to identifying organic remnants, retrieving DNA from human tissues and skeletal remains, and examining genetic factors related to domestication, with significant potential in regions challenged by heat and humidity (Inkret & Pajnič, 2025; Tomei et al., 2023).

However, using biomarkers to identify plant taxa is limited by incomplete understanding of molecular survival, since many compounds decay unless protected by artifact state or depositional context. Hence, biomarker methods rely on linking compounds in archaeological samples to the known chemistry of modern plants (Huber et al., 2022). Material composition can also change during or after storage (Badillo-Sanchez, Ruber, Davies-Barrett, Jones, & Inskip, 2023).

The utilization of genetic analysis to address enduring inquiries in archaeology is increasingly prevalent. Ancient DNA (referred to as aDNA) has demonstrated remarkable preservation in bones, teeth, eggshells, invertebrate shells, coprolites, dental calculus, hair, stone tools, unglazed pottery and plant remains (Chen & Nedoluzhko, 2023; D'Agostino et al., 2023) Over the past decades, aDNA has significantly contributed to understanding evolutionary history and the biological past. For instance, investigations into plant microremains and aDNA preserved in dental calculus have yielded novel insights into historical human diet and health (Tanasi et al., 2021).

Biomolecular techniques widely embraced in archaeology encompass multi-omic approaches, such as genomics, proteomics, lipidomics, and metabolomics. These tools have been developed across disciplines (Badillo-Sanchez, Ruber, Davies-Barrett, Jones, & Inskip, 2023). The fundamental objective of multi-omic studies is to understand interactions among biological systems and facilitate biomarker discovery (Boggi et al., 2023).

The application of diverse techniques has enabled identification of animal products, milk and vegetable lipids, fish and shellfish, as well as fermented beverages such as wine and beer (Tanasi et al., 2021).

Additionally, volatile compounds may evaporate or alter over time due to environmental factors, leading to fragmentation or formation of derivatives common across species (Huber et al., 2022).

The objective of this review is to comprehensively address the latest analytical techniques applied to archarobotanical and archaeological remains reconstruct ancient diets, a key factor in influencing health. It aims to demonstrate the significant capabilities and potential offered by advanced biomolecular techniques, while also highlighting the most pressing challenges that necessitate resolution.

While these approaches have substantially expanded the scope of ancient dietary studies, they remain constrained by preservation biases, contamination risks, methodological variability, and dependence on modern reference datasets. Outcomes can be skewed toward well-preserved or specific compound classes, leaving significant gaps in reconstructing fragile dietary components. The integration of biomolecular data with archaeological and anthropological contexts is essential to avoid over interpretation and to refine narratives of health, subsistence, and human–environment interactions.

## 2 Archaeobotanical and archaeological remnants of interest

**Fig. 1:**
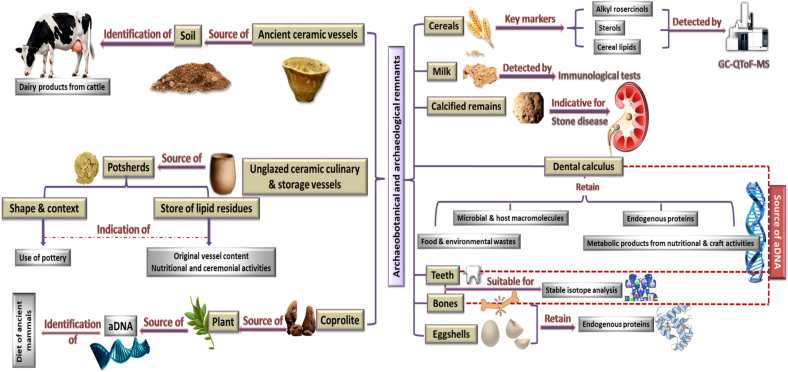
Schematic representation for archaeobotanical and archaeological remnants and their indications for understanding ancient diet and dietary habits

For better understanding, we herein outline the characteristic archaeobotanical and archaeological remnants of interest that will be discussed in this review and are related to the excavated Zaki Saad collection (Fig. 1).

2.1 . Cereals

Cereal-specific compounds like alkylresorcinols, plant sterols as well as cereal lipids should be analyzed using highly sensitive methods based on GC-QToF-MS (Polla & Springer, 2022).

2.2 Milk

Milk residues have been frequently detected in archaeological contexts where immunological tests are employed for their exact determination (Evans et al., 2023).

2.3 Calcified remains, dental calculus, and eggshells

Calcified remnants infrequently retrieved during burial excavations (Ordóñez et al., 2024) confirmed the prevalence of stone disease among the Greek and Roman civilizations in ancient records (Mutlu, 2024).

Dental calculus as well as other substrates viz. bone and eggshell have a greater capacity to retain endogenous proteins compared to others (Tanasi et al., 2021).

Dental calculus analysis can be a rich source of archaeological knowledge since it retains not only microbial and host macromolecules, but also food and environmental waste, as well as metabolic products that are presumably the result of nutritional and craft activity (Rogóż et al., 2021).

Significant research has been conducted on macromolecules such as DNA and proteins found in archaeological teeth, bones, and dental calculus to determine aspects such as historical nutritional consumption, infectious illnesses, genetic heritage, and human bio-social systems (Badillo-Sanchez, Jones, Inskip, & Scheib, 2023).

Stable isotope analysis entails the measurement of isotopic ratios of different elements within human tissues, such as bones or teeth. This method yields valuable insights into the historical diet, weaning stress, migration patterns, and environmental conditions of past populations (Vodanović et al., 2023).

2.4 Soil, Coprolites & Pottery remains

The soil samples from ancient ceramic vessels were examined for the presence of dairy products from cattle (Tarifa-Mateo et al., 2024).

The bulk screening of plants in coprolites offers an insight into the diets of ancient mammals via ancient DNA analysis (Johnson et al., 2024).

Pot sherds, which are fragments from unglazed ceramic culinary and storage vessels, are among the most frequently encountered artifacts during archaeological excavations. Aside from the chronological and other information derived from obvious aspects of these sherds, they also carry secret chemical information indicating their usage history. The shape, archaeological context, and associations with other items are key factors in determining the uses of pottery (D'Agostino et al., 2023). Lipid residues (and other dietary elements) can be stored for millennia by being absorbed in the inorganic matrix and shielded against microbial breakdown and water leaching. These accumulated lipid residues are a valuable source of knowledge because they allow reconstruction of the original vessel contents, as well as the nutritional, ceremonial, and food procurement activities of previous cultures (Korf et al., 2020).

## 3 Inorganic isotope analysis

Isotopes are distinct forms of the same chemical element characterized by an identical number of protons—defining the element—while differing in their number of neutrons. They are classified into unstable (radioactive) isotopes and stable (non-radioactive) isotopes. Unstable isotopes spontaneously decay, emitting various forms of radiation in the process. In contrast, stable isotopes do not undergo decay over geological timescales. This renders stable isotopes particularly valuable as physiological and environmental tracers (Ferrio et al., 2020).

Several isotopes are considered of interest in archaeology such as the stable isotopes ^13^C, ^15^N, ^34^S, ^18^O (Somerville & Beasley, 2023), and ^2^H (Sharpe & Krigbaum, 2022). Other important isotopes used are radiogenic ^87^Sr and non-radiogenic ^86^Sr where the ^87^Sr/^86^Sr ratio gives valuable information about migration or residential mobility (Somerville & Beasley, 2023) in addition to Zn isotope ratios (^66^Zn/^64^Zn) which are considered non-traditional stable isotopes used, among other types of isotopes as an important dietary indicator present in the enamel bioapatite as well as bones (Bourgon et al., 2020; McCormack et al., 2021, McCormack et al., 2022)*.*

Stable isotope analysis entails the measurement of isotopic ratios of different elements within human tissues, such as bones or teeth. This method yields valuable insights into the historical diet, weaning stress, migration patterns, and environmental conditions of past populations (Vodanović et al., 2023). Within this framework, the carbon isotope composition of archaeobotanical remains can offer insights into environmental conditions, agricultural practices, food networks, and dietary patterns, among other aspects (Bernardini et al., 2025; Ferrio et al., 2020; Vilanova & Porcar, 2020).

Isotope analysis is based on measuring the ratio or the relative abundance of isotopes after undergoing disintegration, which is dependent on the nature of the isotope, in addition to certain other factors, both physical and chemical. One of the most important isotopes used is carbon-13, which is a major constituent of organic matter and thus can be used as a tracer for various physiological and environmental processes in plants. Other isotopes of interest include oxygen, which gives important data concerning climate and seasonality, strontium, which gives important information related to geology and animal diet and others like nitrogen which is also related to animal diet. An important note is the combined use of isotope analysis to give a holistic idea concerning an archaeological time period (Ferrio et al., 2020; Madgwick et al., 2021). Despite its utmost importance, its results must be subjected to extensive scrutiny to avoid bias and misinterpretations, ultimately leading to false or otherwise inaccurate results (Madgwick et al., 2021). Isotope analysis has been pivotal in revealing the agricultural practices (Ros et al., 2024), and monitoring changes in climate (Hart et al., 2022). Crucial information have been extracted from isotope analysis of charred plant remains such as crop deposition and crop growing conditions (Baumann et al., 2025; Nitsch et al., 2019), chronology of crops in different cultures, diet and consumption of crops (Soncin et al., 2025) and manuring practices (Schlütz et al., 2025). Examinations of stable isotopes found in plant remains from Arslantepe (Turkey) suggested considerable climatic variations during the transition from the 4th to the 3rd millennium BCE and indicated that irrigation techniques were introduced in response to these changing environmental conditions (Frangipane, Di Nocera and Restelli, 2021).

**Fig. 2:**
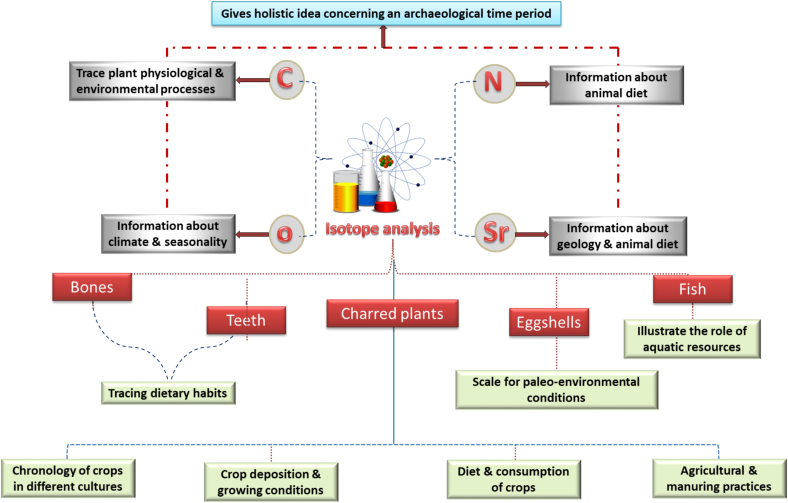
Schematic representation for applications of isotope analysis in archaeobotanical and archaeological remnants.

Isotope analysis is also used for other types of samples. For example, isotope analysis of teeth and also bones has proven crucial in tracing dietary habits of the population (Eerkens et al., 2024). In addition, ostrich eggshells have been successfully examined using isotope analysis and used as a scale for paleoenvironmental conditions (Niespolo et al., 2020). Another research study made use of the technique in describing the importance of Rheidae eggs in the human diet (Abbona et al., 2024). Fish analysis was also possible using the technique to illustrate the role of aquatic resources affecting ancient economy (Fontanals-Coll et al., 2023) (Fig. 2).

Isotope analysis has been of immense importance and is considered an indispensable tool in archaeological studies. However, a cautionary note to be taken is that most studies are usually conducted locally, i.e. in a limited time span and relatively constrained geographical site, thus leading to different challenges arisen such as the small quantity of studied isotopes, the difficulty of using the conducted studies comparatively in addition to other factors interfering with isotopes concentrations. As a result, it is advised to use a combination of osteological, archaeological and environmental data for obtaining correct results within the archaeological framework (Pollard et al., 2023).

Numerous techniques are used for the isotope analysis and a wealth of literature is published covering different techniques such as mass spectrometry, x-ray fluorescence spectroscopy (Haschke et al., 2021; Shackley, 2022; Zheng et al., 2019). Subsequent sections are dedicated to two of the most important techniques for isotope analysis.

### 3.1 Inductively coupled plasma mass spectrometry (ICP-MS)

ICP-MS, first introduced in the 1980s, is now considered one of the most important element-specific detection techniques. Similar to other mass spectrometric techniques in principle, the ion source is, however, a high temperature plasma discharge that is suitable for detecting most elements present in the periodic table (Clases, Gonzalez and de Vega., 2022). The technique also enjoys different sample introduction techniques and, upon hyphenation with other separation techniques such as GC, it has grown in importance even more to become one of the most versatile techniques present (Suárez-Criado et al., 2022).

ICP is an attractive tool in archaeology with several applications such as rocks chemical analysis (de Moraes et al., 2022), elemental concentrations in crops (Blanz et al., 2019), analysis of elements in bones, teeth, and enamel (Dussubieux, 2020; Nesmiyan et al., 2024), analysis of soils and sediments (Janovský et al., 2024; Mira et al., 2024), analysis of peat (partially decayed vegetation or organic matter) (Segnana et al., 2020), analysis of floor samples (Wyżgoł et al., 2024) and determination of isotope ratios (Lin et al., 2022).

### 3.2 Neutron activation analysis (NAA)

Neutron activation analysis is, as the name suggests, a technique of measuring the concentrations of different elements by bombarding stable nuclei with neutrons to convert them to other radioactive nuclei via nuclear reactions and then measuring the resultant radiation. Despite being a very attractive technique due to its versatility and non-destructive nature, a major drawback is present in the principle of the technique itself which is the use of nuclear reactors to achieve the required result in addition to the high resulting radioactivity limiting its use in archeometry (Ghanem et al., 2024; Riehle et al., 2023). The technique has found different interesting applications in different fields as well, in addition to archaeology (Cazzaniga et al., 2021; Glascock, 2022).

## 4 Organic residue analysis (ORA)

Natural products discovered and utilized in archaeological contexts typically consist of a combination of both organic and inorganic compounds (Hristova et al., 2024). Residue analysis in the field of archaeology entails the examination of organic remnants from historical periods through the utilization of advanced instrumental analytical chemical and biochemical techniques which necessitate a high degree of selectivity and sensitivity, since the components of organic residues typically occur at trace concentrations. These organic residues are typically found bound to, adhered to, or absorbed within mineral artifacts such as ceramic vessels, human or animal remains, soils/sediments, or stone tools. Analysis methods encompass a wide range, from the microscopic identification of residual tissue fragments to the chemical and structural analysis of key biomolecules, including lipids, proteins, and DNA (Badillo-Sanchez, Ruber, Davies-Barrett, Jones, & Inskip, 2023; Craig et al., 2020; Hendy, 2021; Irto et al., 2022).

The archaeological data embedded within organic residues is delineated by the biomolecular constituents of natural products that contribute to the composition of a specific residue (Craig et al., 2020). Hence, detailed assessment of the assortment and the level of chemical modifications as well as the susceptibility to microbiological attacks, pH, redox potential, temperature, wetness (arid versus waterlogged), and biomass effects is crucial for maximizing their recovery and authenticity, therefore offering valuable information concerning previous cultural and economic endeavors (Broda et al., 2023; Irto et al., 2022; Lodwick & Rowan, 2022).

## 5 Sample selection and preconditioning

Prior to organic residue bioarcheological analysis, the most critical issues to be considered include whether the applied technique is destructive or not. Consequently, samples should be categorized according to their status; either they should be fully conserved or we can apply micro-invasive techniques (Polla & Springer, 2022).

Thorough and precise documentation of the archaeological context of the findings viz. climatic conditions, probable sources of contamination or adulteration, besides proper treatment of the whole pathway of the find, guarantees a successful analysis of the organic content of archaeological artifacts (Polla & Springer, 2022).

## 6 Multi-Omics

In contrast to traditional biomolecular methods, the multi-omics technologies allow for the high-throughput analysis of biological data in genomics, proteomics, metabolomics, and lipidomics in one experiment, hence simultaneously providing a comprehensive dataset on a wide range of biomolecules present in a sample. The primary advantages of omic technologies for archaeological research lie in their untargeted nature and their ability to generate a large amount of data in a short time (Badillo-Sanchez, Jones, Inskip, & Scheib, 2023; Procopio & Bonicelli, 2024; Vilanova & Porcar, 2020).

The emergence of cost-efficient, high-performance technical platforms such as genetic sequencers, ultra-high performance liquid chromatography (UHPLC) and mass spectrometers paved out the basis for the study of omics disciplines, typically known as ‘technology-based omics’ (Procopio & Bonicelli, 2024).

Furthermore, transformative advances in data processing and informatics have resulted in better precision and accuracy of the instruments, allowing for the identification of a broader repertory of molecules with low (or ultralow) concentration yet can be recognized with higher certainty (Badillo-Sanchez, Ruber, Davies-Barrett, Sandhu, Jones, et al., 2023).

**Fig. 3:**
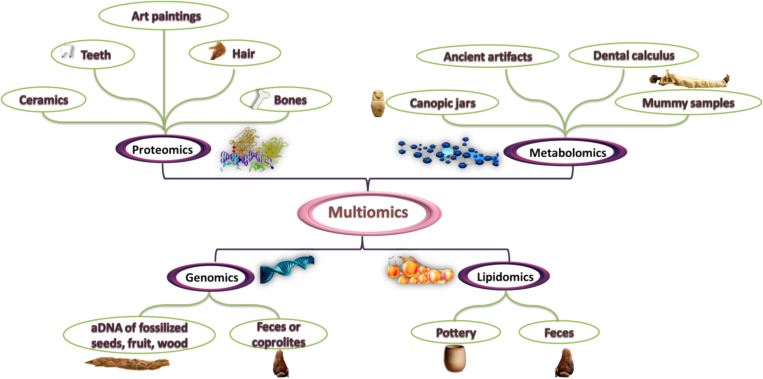
Applications of Omics techniques in the analysis of archaeobotanical & archaeological remains

Integration between omic-technologies viz. metabolomics and lipidomics might help to investigate aspects that genomes and transcriptomics cannot explain, such as environment or diet (Stepanova, 2019) (Fig. 3).

### 6.1 Genomics

Genomics is the analysis of DNA to understand the genetic makeup of an organism, which in bioarchaeology has become crucial as it enables researchers to track the lineage and connections of individuals from past societies (Orlando et al., 2021). In archaeobotanical research, the primary source of ancient plant DNA has been archaeobotanical remains, including fossilized seeds, fruit, and wood (Kistler et al., 2020). Desiccated feces, or coprolites, can undergo genetic analysis to determine the organism that produced them and the plants and animals they consumed. The bulk screening of plants in coprolites for aDNA studies offers an intriguing chance to gain insight into the diets of ancient mammals (Witt et al., 2021). This genomic approach complements traditional biomolecular methods by providing a deeper, molecular-level understanding of plant history and human-plant interactions.

#### 6.1.1 Genomics as a complement to traditional archaeobotany

Genomic analysis allows researchers to look beyond the physical form of a plant and into its genetic code, where the true history of domestication is written. For example, by comparing the aDNA of a crop from different archaeological sites, scientists can identify shared genetic mutations linked to traits desirable for agriculture, such as seed size, non-shattering heads, or disease resistance (Zhang et al., 2022). This genetic data can validate morphological observations. If archaeobotanists find that ancient wheat seeds from a site in the fertile crescent are larger than their wild ancestors, genomic analysis can confirm that this change was due to human selection by identifying the specific genes responsible for increased seed size (Haas et al., 2019). Furthermore, genomics can refine our understanding of crop migration patterns. Traditional methods might show a crop appearing in a new region, but it's often unclear if it was introduced through a single event or multiple, separate introductions. Genetic analysis can reveal the pathways of these migrations (Kistler et al., 2020; Pont et al., 2019; Xiong et al., 2025). In essence, while traditional archaeobotany provides the “what” and “where,” genomics provides the “how” and “when” on a molecular level, offering a richer, more nuanced narrative of human-plant co-evolution.

#### 6.1.2 Archaeological findings derived from genomic analysis

aDNA analysis is utilized to resolve a variety of complex questions concerning crops origin, migration routes, and even the historical dissemination of infectious diseases. Recent genomic studies in archaeobotany have provided unprecedented insights into the history of plant domestication, evolution, and migration. For instance, genomic analysis of a 2100-year-old date palm leaf from Saqqara, Egypt, traced the plant's ancestry to modern domesticated North African date palms and their wild relatives, providing a new timestamp on the domestication of this crop (Pérez-Escobar et al., 2020). Additionally, aDNA analysis of charred seeds from Neolithic sites in Anatolia helped confirm the presence and identity of *Triticum aestivum* subsp. *spelta* (spelt wheat), shedding light on the domestication and dispersal routes of this ancient crop (Değirmenci et al., 2023). Genomic investigation of ancient cotton remains, particularly from sites like Qasr Ibrim, assisted in reconstructing the evolution of Cotton. This helped us understand the evolutionary relationship between Old World and New World cotton varieties and the processes that shaped their domestication (Viot & Wendel, 2023).

Researchers utilized whole-genome sequencing of over 1400 rice landraces to map the history of rice dispersal in Asia which revealed that rice originated in the Yangtze Valley about 9000 years ago which later diversified into two subspecies—*japonica* and *indica*—in response to climatic factors. During the global cooling event 4200 years ago, temperate and tropical *japonica* rice have emerged. This comprehensive reconstruction of rice's spread and its connection to past climates provides valuable insights into the genetic adaptations that enabled this vital crop to thrive across a vast region (Gutaker et al., 2020). Further analysis demonstrated that *japonica* rice in the northern Philippines diverged from Indonesian landraces around 3500 years ago (Alam et al., 2021). A key step in rice domestication is losing the ability to drop its seeds in a process called shattering. While a single gene mutation (*sh4*) was once thought to be responsible for this, a study published in 2022 found that it's not enough on its own. Other genes, like *qSH3*, are also needed to completely stop the seeds from falling off (Ishikawa et al., 2022).

In case of legumes, several researches show that they were domesticated in several global regions, forming a cornerstone of early agricultural societies. The loss of seed dispersal mechanisms is a key domestication trait in many legumes. A recent study investigated this phenomenon in the adzuki bean, *Vigna angularis*, and the yard-long bean, *Vigna unguiculata*. Using fine-mapping techniques on backcrossed populations, researchers successfully narrowed down the responsible genomic region to a very small area. Additionally, a specific gene, MYB26, was found to be responsible for their pod shattering. Hence, MYB26 could serve as a valuable target gene to improving shattering traits in other legume crops, such as soybeans (Kachapila et al., 2023; Takahashi et al., 2020; Zhang et al., 2022). A study analyzing ancient and modern common bean *Phaseolus vulgaris* seeds from the southern Andes investigated the temporal dynamics of genetic variation and selection during its domestication. The findings revealed that most domestication traits were selected for over 2500 years ago with minimal loss of overall genetic heterozygosity. The ancient bean genomes, dating between 600 and 2500 years ago, were found to be highly variable—comparable to modern wild populations. The research indicates that while modern cultivars show reduced genetic variation per seed, the pooled genomic diversity of different cultivars from various Andean regions is higher than that found in ancient seeds from the same areas (Trucchi et al., 2021).

A comprehensive genetic analysis of over 3500 wild and cultivated grape varieties has provided new insights into their domestication history. Previously, the exact origins of wine and table grapes were unclear due to incomplete data. However, several new studies found that both wine and table grapes were likely domesticated at the same time and that past climate changes influenced the size of grape populations. This work significantly improved our understanding of how both humans and the environment shaped the evolution of this culturally and economically important crop (Dong et al., 2023; Schneider et al., 2024; Zhou et al., 2019). Moreover, Genetic research on ancient watermelon seeds has revealed new insights into their early history. Pérez-Escobar and his colleagues sequenced the genomes of 6000- and 3300-year-old watermelon seeds from Libya and Sudan, along with DNA from a vast collection of modern and historical samples. Their analysis showed that the ancient seeds' DNA was more similar to the West African “egusi-type” watermelon, which is primarily grown for its seeds, rather than the modern sweet-fleshed watermelon. Based on the presence of specific genes that control bitterness and flesh color, the 6000-year-old watermelon likely had a bitter and greenish-white flesh. Additionally, both ancient genomes showed a mix of DNA from several different watermelon species, indicating a complex history of interbreeding (Pérez-Escobar et al., 2022). Even parasitic plants that can devastate maize yields such as *Striga*, or witchweed was shown to be triggered to germinate by strigolactones released from maize roots. A study by Li et al. discovered that maize varieties that produced zealactol were more resistant to *Striga* infection compared to those that produced zealactone which is catalyzed by cytochrome P450 (Li et al., 2023).

#### 6.1.3 Challenges and limitations in genomics

The application of genomics in aDNA research has extended our understanding of genetic diversity beyond contemporary populations. Rather than being limited to the genetic makeup of modern-day organisms, researchers can now utilize datasets that track the changes in the genetic ancestry of human, animal, and plant even microbial populations. While the exploration of aDNA has yielded remarkable results and is a growing area of study, it remains a field with significant challenges and limitations.

A major characteristic of aDNA is the different types of DNA damage. Fragmentation and deamination of cytosine to uracil are the major mechanisms leading to miscoding lesions in aDNA (Peyrégne & Peter, 2020; Zhao et al., 2025). Therefore, the removal of deaminated cytosine is advantageous to decreasing the sequencing error rate. Treatment of ancient samples with uracil-DNA-glycosylase and endonuclease VIII removes uracil residues and repairs most of the resulting abasic sites while leaving undamaged parts of the aDNA fragments intact. Additionally, oxidation can induce lesions that block polymerases, namely blocking lesions, and stop PCR amplification processes (Psonis et al., 2021).

Contamination of aDNA samples is another limitation that plays a critical role in determining the quality and efficiency of the sequencing process. For example, contamination may occur due to airborne particulates and adherence or uptake of exogenous DNA from microorganisms in the environment surrounding ancient samples such as bones and teeth (Peyrégne & Prüfer, 2020). Contamination may also be introduced during the collection of the samples and other experimental processes, including aDNA extraction and sequencing library preparation, or PCR setup (Peyrégne & Prüfer, 2020). All these factors contribute to the low quality and quantity of endogenous DNA in ancient samples. Therefore, removing such contamination is invaluable for an efficient sequencing process and accurate results (Matsvay et al., 2019).

Another major challenge when performing population genetics and other studies is that a single sample can generate more data than needed from a next-generation sequencing (NGS) instrument, making the cost of analyzing many individual samples too high. To solve this, researchers pool multiple samples together to be sequenced in a single run. Since this process makes it impossible to know which sample a specific DNA sequence came from, barcoding techniques are used. These techniques involve adding a unique genetic tag, or barcode, to the DNA from each sample. After sequencing, these barcodes help bioinformatic tools sort the millions of reads produced and assign them back to their original sample. This approach is essential for making large-scale genomic studies more efficient and affordable (Childebayeva & Zavala, 2023). Even proper archiving of the produced genomic data from aDNA is another challenge. Incomplete archiving of datasets prevents accurate replication of the data leading to their loss, so they can't be potentially used again in the future (Bergström, 2024).

Furthermore, genomic analysis of mitochondrial DNA (mtDNA) from highly degraded samples is extremely challenging. To overcome the challenges of ancient mtDNA analysis, a new method has been developed that includes specific improvements, such as increasing DNA input from low-concentration extractions, using a size-selection step, and monitoring the pre-capture PCR in real time to avoid the need for manual cycle optimization. Such methods aims to be a user-friendly and cost-effective solution for analyzing many aDNA samples (Senovska et al., 2021). Many researchers currently focus on the limitations and challenges of aDNA genomic analysis in order to provide solutions and improve aDNA sequencing procedures.

6.1 Metabolomics

The primary focus of metabolomics lies in the qualitative and quantitative characterization of small (endogenous) molecules (<1500 Da) and subsequent comprehensive multivariate statistical analysis to discern between two sample classes. This entails leveraging the entirety of the chemical composition data, including positive hits and non-identified features, to identify potential similarities and variances in the sample classes (Bonicelli et al., 2024).

Metabolomics approach enables the evaluation of metabolomic profiles and metabolic pathways, identification of biomarkers for particular conditions, and comparison of phenotypic differences among populations (Badillo-Sanchez, Ruber, Davies-Barrett, Jones, & Inskip, 2023).

Being advanced, sensitive, and reliable besides utilizing low mass for analysis of samples, metabolomics is becoming a more viable option with tremendous potential in the archeological sector, where the study of metabolomics of different organic and inorganic archaeological residues provides integrative overview of conditions in the past (Badillo-Sanchez, Ruber, Davies-Barrett, Sandhu, Jones, et al., 2023; Badillo-Sanchez, Ruber, Davies-Barrett, Jones, & Inskip, 2023).

Small molecules, especially secondary metabolites like phenolics, alkaloids, benzenoids, and terpenes, are crucial targets for plant identification. For instance, terpenoids in plant resins or alkaloids in psychoactive plants are sometimes source diagnostic, allowing for the identification of botanical genera and even species when compared to modern plants (Huber et al., 2022).

Untargeted metabolomics has been used to chemically characterize the content of canopic jars and mummy samples from Ancient Egypt, resulting in the discovery of thousands of ancient molecules. Nevertheless, specific embalming recipes for individual samples or organs were not identified (Barberis, 2020).

Metabolomics have been employed in archaeological context to investigate dental calculus and ancient artifacts in order to determine the usage of alkaloids by prior societies (Badillo-Sanchez, Ruber, Davies-Barrett, Sandhu, Jones, et al., 2023).

Metabolomic approaches are still not strongly applied in the archaeobotanical context identification (Badillo-Sanchez, Ruber, Davies-Barrett, Jones, Hansen, & Inskip, 2023).

### 6.2 Lipidomics

Organic residues commonly found in archaeological contexts are often lipids. Lipids are organic compounds that have low solubility in water (Eusebio, 2019; Gerhardtova et al., 2024) and are anticipated to exhibit relatively superior preservation in comparison to carbohydrates, proteins, and nucleotides. Their inherent resistance to decay due to their low levels of functional groups and the prevalence of saturated aliphatic chains, branches, and rings in their structure, coupled with their likelihood of enduring at the original deposition site due to their hydrophobic properties, renders them highly suitable for implementation as biomarkers in archaeological inquiries (Harrault et al., 2022; Vilanova & Porcar, 2020). Hence, lipids are the most frequently found organic compounds in pottery due to their high resistance to deterioration. Moreover, lipids are highly retained in charred surface residues, likely because microencapsulation inhibits microbial activity (D'Agostino et al., 2023; Irto et al., 2022).

The classification of lipids is crucial due to their large number. Lipids can be categorized into two groups based on their polarity: those that become water-soluble upon saponification or alkaline hydrolysis (such as oils, fats, and waxes) and those that do not (like resins and sterols) (Eusebio, 2019; Gerhardtova et al., 2024). From the perspective of organic geochemistry, lipid biomarkers are classified as isoprenoids (steroids, hopanoids, and terpenoids), long-chain waxes (plant waxes and beeswax), and acylglycerides and fatty acids (plant oils, animal adipose fats, and aquatic biomarkers). In archaeological studies, lipid biomarkers are useful for distinguishing between major food types, including terrestrial meat, aquatic resources, and plants (Eusebio, 2019). Lipid extracts can provide valuable insights about dietary constituents and manufacturing processes (Mallol et al., 2025).

Lipidomics relies on advanced high-resolution instruments and specific data-processing methods for investigating entire lipid assemblages instead of individual compounds. For instance, lipidomics can uncover the significance of low-abundance compounds in understanding biochemical or biosynthetic connections between compounds or in discovering biomarkers (Wei et al., 2025).

Over the past five decades, there has been a remarkable proliferation in the utilization of ancient lipid analysis. This has encompassed a wide array of applications, including inquiries into the dietary habits of prehistoric societies, agricultural practices, and various other cultural and societal endeavors of antiquity (Behera & Gadekar, 2025).

For instance, lipid biomolecules viz. n-alkanes, sterols, phytosterols, bile acids, and lignin may be examined to establish the biogenic origin of ancient excrement and, consequently, the existence of prior animal husbandry as well as determining the diet of past populations (Florenzano et al., 2023; Vallejo et al., 2022). The detection of biomarkers viz. sterols, phytosterols (of plant provenance) and bile acids in dung might help reveal the biogenic origin of the corresponding substances, either human, canine, herbivore, porcine, or ruminant (Vallejo et al., 2022).

Terpenoids from resinous plants are the most well-preserved plant biomolecules. For instance, the detection of benz[*a*]anthracene, a type of polynuclear aromatic hydrocarbon, signifies the occurrence of wood combustion in an open fire. Additionally, the identification of hexyl-cinnamaldehyde suggests the potential processing of plant material, likely originating from the Asteraceae family (Eusebio, 2019).

Despite their abundance in most food lipids, unsaturated fatty acids are highly vulnerable to oxidative degradation, hence rarely recovered. Hence, the degradation products of unsaturated fatty acids are used to conclude the authentic existence of such lipids. Likewise, palmitic and stearic acids the hydrolytic products of ester lipids viz. triacylglycerols & wax esters predominate the recovered lipids from archaeological pottery matrices (Korf et al., 2020).

Recently, gas chromatography combined with mass spectrometry (GC–MS), gas chromatography-isotope ratio mass spectrometry (GCIRMS), high-performance liquid chromatography (HPLC), high-performance liquid chromatography coupled with atmospheric pressure chemical ionization-mass spectrometry (HPLC–APCI-MS), and high-performance liquid chromatography combined with electrospray ionization-quadrupole time of flight mass spectrometry (HPLC-ESI-QToF) have been utilized for lipid analysis in archaeometry (Manzano et al., 2019).

6.2 Proteomics

Proteomics is a cutting-edge method interested in the study of the group of proteins molecules an organism expresses, namely proteome. Employing this technology in the study of cultural heritage materials allows for the identification of proteins in archaeological context viz. bones or hair samples, hence enabling the reconstruction of the diet and health of ancient populations through the combination of chromatography and mass spectrometry (Elnaggar et al., 2022; Vodanović et al., 2023).

The study of ancient samples using traditional proteomic methods is the goal of paleoproteomics, a burgeoning field in mass spectrometry which is expanding and utilizes liquid chromatography–tandem mass spectrometry (LC–MS/MS) methods, also known as “shotgun” approaches, to potentially identify food items prepared in ceramics (Tanasi et al., 2021; Warinner, Richter and Collins, 2022). Paleoproteomics also involves studying a group of proteins to determine the species and evolutionary connections of extinct animals (Elnaggar et al., 2022).

The presence of ancient proteins is widespread in preserved human and animal mummies, as well as in materials made from processed skin and hair, like parchment. Most ancient protein research is based on bones and teeth. Analyzing ancient bone proteomes has become common practice, with collagen type 1 being the most prevalent component of the bone extracellular matrix. However, the study of ancient proteins from plant remains has been infrequent until now (Evans, 2025; Hendy, 2021; Procopio et al., 2021).

Application of proteomics to ancient art paintings aimed at identifying the protein-based substances used to prepare the pigments. There are two main methods for conducting these studies: (i) untargeted LC–MS/MS, which aims to identify a wide range of peptides in the sample, and (ii) MRM (multiple reaction monitoring), which focuses on specific peptides known to be indicative of various potential binders such as casein (a clear marker for milk), ovoalbumin (egg), or vitellogenin (yolk) peptides. Proteomics has also been crucial in revealing the composition of food remnants discovered in ancient ceramic vessels, providing valuable insights into the lifestyle and customs of prehistoric societies (Vilanova & Porcar, 2020).

While mass spectrometry has been successful in identifying ancient proteins in bones and tissues, there have been fewer instances of recovering and identifying protein residues from ceramic artifacts due to the limited presence of proteinaceous materials. Studies on protein residues from ceramics revealed a complex and tiered mixture of protein residues left behind since pottery was frequently reused for various food preparation uses (Tanasi et al., 2021).

## 7 Analytical platforms

**Fig. 4:**
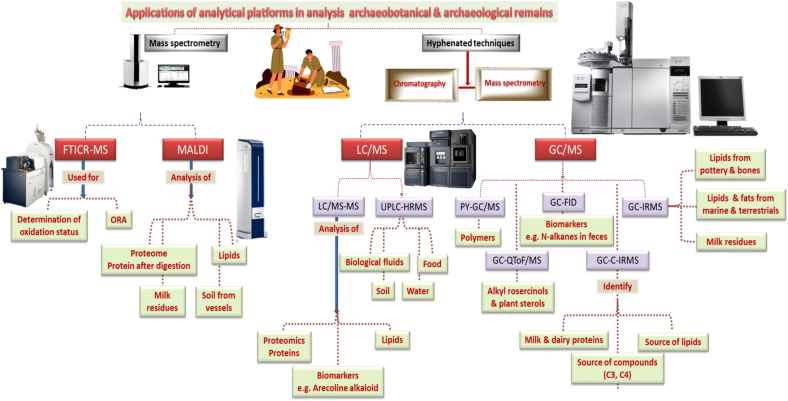
Schematic representation for applications of analytical platforms in the analysis of archaeobotanical & archaeological remains

Different analytical platforms are employed for the analysis of archaeobotanical and archaeological remnants and will be discussed in details in the next subsections (Table 1, Fig. 4).

7.1 Mass spectrometry

**Table 1:** Comparison between different analytical techniques employed for analysis of archaeobotanical and archaeological remnants.

Residue analyzed	Technique		Associated omics	Applications	Advantages	Disadvantages	References
Inorganic	Neutron activation analysis (NAA)		–	Measuring the concentrations of different elements in archaeology	Versatile; Non-destructive	Use of nuclear reactors; high resulting radioactivity	(Cazzaniga et al., 2021; Ghanem et al., 2024; Glascock, 2022; Riehle et al., 2023)
Inorganic	Mass spectroscopy	Inductively coupled plasma mass spectrometry)ICP-MS)	–	Analysis of: rocks; elemental concentrations in crops; elements in bones, teeth and enamel; soils and sediments; peat (partially decayed vegetation or organic matter); floor samples; determination of isotope ratios	Suitable for detecting most elements present in the periodic table	Detector noise; can't resolve most isotopes	(Blanz et al. Stepanova, 2019; Clases, Gonzalez and de Vega., 2022; Dussubieux, 2020; Janovský et al., 2024; Lin et al., 2022; Mira et al., 2024; de Moraes et al., 2022; Nesmiyan et al., 2024; Segnana et al., 2020; Wyżgoł et al., 2024),
Organic		Fourier transform ion cyclotron resonance mass spectrometry)FTICR MS)	Metabolomics	Analysis of higher-mass compounds; determination of the oxidation state of the sample	Ultra-high-resolution & high-mass accuracy; quick and comprehensive characterization of organic residues	Poor detection sensitivity due to ion suppression effect	(Gosset-Erard et al., 2023; Hertzog et al., 2023).
			Lipidomics	Characterization of lipids and triacylglycerols			
			Proteomics	Characterization of proteins			
		Matrix assisted laser desorption/ionization)MALDI)	Lipidomics	Identification of lipid residues	Soft ionization & highly sensitive method combined with FT-ICR-MS	Background matrix ions for small compounds (< 1000 Da);sample photodegradation; in-source dissociations of analytes	(Chowdhury et al., 2021;Drieu et al., 2025;Vykukal et al., 2024)
			Proteomics	Proteome study of organic residues, analysis of milk residues & proteins			
			Metabolomics	Non-targeted analysis of the soil content			
	Gas chromatography-mass spectrometry)GC–MS)		Lipidomics	Analysis of organic remains, mainly lipids	Robust and reliable; high level of certainty	Suitable for volatile compounds; derivatization required	(Hertzog et al., 2023; Korf et al., 2020; Polla & Springer, 2022)
	Liquid chromatography-mass spectrometry (LC-MS)		Metabolomics	Separation & identification of compounds with excellent sensitivity and specificity	Suitable for non-volatile compounds specially, polar to ionic	Mobile phases incompatible with MS detectors; sample preparation	(Beccaria & Cabooter, 2020; Polla & Springer, 2022)

Mass spectrometry is a crucial method in archaeological research that allows for precise identification of trace levels of ancient compounds isolated from microsamples via mass spectrometry. It enables the detection of proteins and lipids in the bones of ancient animals, as well as the examination of dental pulp specimens (Barberis, 2020; Kostyukevich et al., 2019). Bitumen and resin, commonly found in pottery vessels during archaeological excavations, can also be analyzed using modern mass spectrometry to determine their source and origin. Mass spectrometry has also advanced the study of the history of brewing on the molecular level, as demonstrated in the literature (Kostyukevich et al., 2019).

#### 7.1.1 Fourier transform ion cyclotron resonance mass spectrometry (FTICR MS)

To analyze higher-mass compounds and unravel the molecular complexity of archaeological organic residues, ultra-high-resolution & high-mass accuracy mass spectrometers, specifically Fourier transform ion cyclotron resonance mass spectrometry (FTICR MS) are employed (Hertzog et al., 2023).

FTICR MS through direct infusion is valuable for quickly and comprehensively characterizing organic residues in archaeological samples and can provide guidance on determining the oxidation state of the sample. However, the application of this technique has been used in a limited number of studies to analyze archaeological samples and characterize lipids, triacylglycerols, and proteins (Hertzog et al., 2023).

#### 7.1.2 Matrix assisted laser desorption/ionization (MALDI)

Matrix assisted laser desorption/ionization (MALDI) is a soft ionization method that generates ions directly from solid samples. The analyte is ionized by the laser with the help of a matrix material, which absorbs radiation at the laser wavelength and is present in large excess compared to the substance being analyzed. The matrix absorbs the laser energy, not the analyte, resulting in the ionization of whole analyte molecules with the addition of a proton or a single positively charged metal ion, usually resulting in H+ or Na + adducts. While MALDI has typically been paired with time-of-flight mass spectrometry (TOF-MS) or Fourier transform mass spectrometry (FTMS), combining it with FT-ICR-MS results in a highly sensitive method that has potential for precisely identifying archaeological lipid residues via detection of larger molecules, viz. triacyl glycerols (TAGs) with more than 54 acyl carbons with a low limit of detection, however not widely used (Drieu et al., 2025; Vykukal et al., 2024). Further, MALDI was utilized in the proteomic examination of ceramic fragments from Tell Khaiber, an archaeological site dating back to the mid-second millennium BCE, as part of a combined methodology with LC-MS/MS to analyze absorbed ancient proteins. Protein residues were prepared for analysis through trypsin digestion, which enabled the identification of milk proteins alongside plant-based proteins like soybean. The integration of MALDI with multivariate data analysis allowed for the non-targeted characterization of protein residues found in the intricate extracts of soil and ceramics. This method improved the sensitivity and specificity in detecting ancient dietary elements, thereby enhancing the understanding of food production and consumption during the Sealand Dynasty period (Chowdhury et al., 2021).

### 7.2 Gas chromatography-mass spectrometry (GC–MS)

Gas chromatography (GC) is among the most robust and reliable widespread analytical techniques which allows for a high level of certainty via comparing peak signals of known markers in the archaeological samples (Polla & Springer, 2022). Coupling of gas chromatography to mass spectrometry (GC–MS) is the most widely adopted technique for analyzing organic remains in archaeological context, notably lipids; however, it is primarily suitable for volatile compounds, and often requires a derivatization step before analysis (Hertzog et al., 2023; Korf et al., 2020).

#### 7.2.1 Applications to lipids and food residues

For instance, GC–MS was employed for the analysis of dairy products, cereals, and animal fat in vessels from the late Bronze Age (Manzano et al., 2019). GC–MS has identified miliacin, a specific fatty acid biomarker for broomcorn millet consumption in North-eastern Medieval Italy. Utilizing a biomarker strategy to identify secondary lipid metabolites from ergot fungi (*Claviceps* genus) helped in recognizing the presence of *Gramineae* and indirectly confirming the utilization of vessels for cereal storage/processing. The presence of certain compounds found in plant products like wheat and rye bran in a wooden container from Switzerland could indicate the presence of biomarkers, supported by macrobotanical remains.

Compound-specific analysis using GC–MS coupled to combustion isotope ratio mass spectrometry detector (GC-C-IRMS) can determine if the compounds come from C3 or C4 plants (Polla & Springer, 2022).

GC–MS and isotope-ratio mass spectrometers (IR-MS) were employed for the analysis of ancient lipids from pottery vessels and bones to uncover the dietary habits of ancient humans (Kostyukevich et al., 2019). Where the stable carbon isotope ratio of palmitic and stearic acids the predominant recovered hydrolytic products of ester lipids from archaeological pottery matrices vary among different species (Korf et al., 2020).

GC-IRMS has also been employed to analyze lipids and fats derived from both marine and terrestrial organisms (Polla & Springer, 2022).

GC-C-IRMS, can also be utilized to differentiate δ13C values, hence distinguishing lipids derived from pig and cow adipose tissues or dairy products (Korf et al., 2020).

The detection of milk residues in archaeological contexts was already described by many authors. The evidence of storage of milk products adherent to the surface of a ceramic vessel or soaked into ceramics from different ages was described by using GC–C–IRMS (Murakami et al., 2022).

#### 7.2.2 Specialized techniques: Py-GC/MS and variants

Among the most common employed techniques for the characterization of embalming fluids/materials is the coupling of a gas chromatograph (GC) to a mass spectrometer (MS) or a tandem mass spectrometer (MS/MS), as well as the more specialized forms of this hyphenated technique viz. sequential thermal desorption-GC–MS or pyrolysis-GC/MS (PY-GC/MS) (Huber et al., 2023; Rageot et al., 2023). PY-GC/MS method enables the generation of specific pyrolysis products from non-volatile samples such as polymers, which can then be separated and examined using GC–MS (Polla & Springer, 2022).

The untargeted PY-GC/MS metabolomics methodology provides a breakthrough and more systematic tool for studying the aging process of multicomponent organic polymers in ancient artifacts (Xingping et al., 2024).

Analyzing lignin, a useful biomarker for determining plant origin and the degradation level of organic matter, is often done through Py-GC/MS, a method that breaks down the natural polymer and identifies all the individual monomers. Every plant possesses its unique lignin monomer composition. Consequently, by examining the lignin, it is possible to ascertain the plant's origin. (Vallejo et al., 2022).

#### 7.2.3 Plant biomarkers and cereal analysis

For the targeted analysis of archaeobotanical cereal biomarkers GC coupled to a high-resolution quadrupole-time-of-flight mass spectrometer (GC-Q-TOF MS) is employed, whereas the analysis of trimethylsilylated lipid extracts is performed by GC coupled to flame ionization detector (GC-FID) and GC-Q-TOF MS (Korf et al., 2020). N-alkanes are effective biomarkers for assessing agricultural and livestock activities. Where, analyzing the ratio of n-alkanes in feces allows for the identification of the plant that was consumed as herbivores cannot readily digest these molecules. This approach can be applied by using GC–MS, GC-FID, or GC-C-IRMS (Vallejo et al., 2022).

Cereal lipids were reported to be detected through cooking experiments and analyzing cereal-specific compounds like alkylresorcinols and plant sterols using highly sensitive methods based on GC-QToF-MS (Polla & Springer, 2022).

#### 7.2.4 Metabolomics and broader applications

Metabolomics has been used to analyze lipids and secondary metabolites, employing GC–MS to examine the composition of art elements and prehistoric objects beyond their protein binders. For instance, microsamples of paint from various artworks revealed the presence of linseed oil, beeswax, and *Pinaceae* resin in the paint mixture. However, the most valuable insights from GC–MS studies have come from the analysis of ancient pottery and other archaeological artifacts. The oldest objects subjected to this method are pottery elements from a cave in China, dating back approximately 20,000–19,000 years BP. The lipid composition of these artifacts, rich in markers dominated by medium- and long-chain saturated and monounsaturated fatty acids as well as isoprenoid fatty acids, indicated the use and storage of high-trophic-level aquatic food (Vilanova & Porcar, 2020).

#### 7.2.5 Dairy and protein studies

Proteomics techniques have recently integrated GC–MS and stable isotopic methods to analyze visible encrustations for more accurate identification of milk and dairy proteins in Neolithic cooking vessels from Çatalhöyük in Turkey (Polla & Springer, 2022).

The analysis of lipids in archaeological ceramics through chromatography generally employs GC–MS for identifying compounds and GC–C-IRMS for isotopic fingerprinting of the C16:0 and C18:0 fatty acids. When used in conjunction, these techniques enable researchers to differentiate between milk fats, body fats, and aquatic resources present in ancient pottery (Rosiak et al., 2025).

#### 7.2.6 Case studies: Diet and consumption practices

In a study conducted by Rogóż et al., GC–MS was adopted for the analysis of a collection of ancient dental calculus taken from the front teeth of the mandibles of seven buried individuals dating back to the 17th- and 18th-century in Southeastern Poland. The analysis revealed the presence of solely saturated fatty acids, which are indicative of animal-derived fats, suggesting that the diet of the inhabitants of this area during the modern period was abundant in animal-based products, including meat and dairy items (Rogóż et al., 2021).

In Northern Vietnam, the analysis of residues in the teeth of Bronze Age inhabitants using GC/MS revealed the presence of Tannin compounds (Eusebio, 2019).

Most of what we know of prehistoric wine consumption comes from organic residue analysis of ceramic containers, unlike botanical remains of the grape. Owing to its polarity, tartaric acid, the principal organic acid in grapes, binds to the ceramic matrix, through strong interactions. Hence, the detection of tartaric acid as a potential marker of wine in ancient ceramic containers indicates their usage to transport or store wine. In this context Gas chromatography coupled with mass spectrometry (GC–MS) is employed for detection of organic acids and other wine-related compounds (Blanco-Zubiaguirre et al., 2019; Drieu et al., 2020).

Manzano et al. analyzed the organic residues in archaeological pottery vessels from Spain via gas chromatography–mass spectrometry (GC–MS), gas chromatography-isotope ratio mass spectrometry (GC-CIRMS), and ultra-performance liquid chromatography-high resolution mass spectrometry (UPLC-HRMS) (Manzano et al., 2019).

### 7.3 Liquid chromatography-mass spectrometry (LC-MS)

By hyphenating liquid chromatography and mass spectrometry, the combined advantages of both high separation power and enhanced identification of compounds with excellent sensitivity and specificity were achieved, making the technique the most suitable for non-volatile compounds, especially polar to ionic compounds. Still, the technique suffers from some drawbacks, such as using mobile phases that are sometimes incompatible with MS detectors and required sample preparation steps to avoid matrix effects, especially in large compounds (Beccaria & Cabooter, 2020; Polla & Springer, 2022). However, LC-MS is still underutilized in organic residue analysis in archaeology when compared to its significance in other analytical science domains (Polla & Springer, 2022).

Liquid chromatography as well as gas chromatography coupled to tandem mass spectrometry analysis, i.e. (LC-MS/MS) and (GC–MS/MS), respectively, can offer enhanced sensitivity and precise detection of proteins in complex samples by identifying the sequence of peptide mixtures at a higher resolution. As a result, the fragmentation quality and subsequent manual confirmation are crucial due to the potential for contamination (Elnaggar et al., 2022). When contrasted to GC–MS, LC-MS has the benefit of being able to omit the derivatization phase (Polla & Springer, 2022).

LC-MS techniques include ultra-performance liquid chromatography coupled to high resolution mass spectrometry (UHPLC-HRMS) which requires dramatically minor sample size for analysis and offers better resolution, as well as hydrophilic interaction chromatography (HILIC) offering unique selectivity. The variation in column dimensions viz. micro- and nano-LC column, besides variability of mass ionization techniques e.g. the ESI-source, nano-electrospray ionization (NSI), generates better sensitivities (Polla & Springer, 2022).

UPLC-HRMS is extensively utilized for the identification of organic compounds in various substances like soils, biological fluids, food, water, and more. Despite its benefits and diverse applications, this method has not been fully adapted for analyzing archaeological samples, and there are limited articles addressing the study of biodeterioration in archaeological materials (Manzano et al., 2019).

Alkaloids are basic nitrogen-containing substances found in plants, animals, microorganisms, and marine organisms. LC-MS/MS and LC-high-resolution mass spectrometry (LC-HR-ToF-MS) detected the presence of arecoline, a biomarker alkaloid specific for Areca nut native to South East Asia, in a stained tooth dating back to an Iron Age skeleton (Eusebio, 2019).

### 7.4 Integrated techniques

The utilization of multiple, hyphenated, high throughput techniques for the analysis of ancient biomolecules can offer a more comprehensive overview for addressing intricate biological inquiries compared to relying on individual biomolecular indicators alone (Tomei et al., 2023). For instance, integrated examination of aDNA and proteins of the ancient oral microbiome revealed a detailed taxonomic and functional description, allowing for simultaneous exploration of pathogen behavior, host immunity, and dietary habits in historical communities (Gancz et al., 2023). Hence, this integrative approach allows for a distinctive understanding of past life.

The analysis of bulk isotopes provides limited information about the origin of lipids associated with major food groups viz. plants, aquatic life, and terrestrial animals. Current standard procedures for the analysis of food crusts combine gas chromatography–mass spectrometry (GC–MS) to characterize organic compounds, gas chromatography–combustion–isotope ratio mass spectrometry (GC–C–IRMS) for compound-specific δ^13^C measurements of fatty acids, and elemental analyzer isotope ratio mass spectrometry (EA–IRMS) for bulk isotopic analysis of residues (Teetaert et al., 2024).

## 8 Limitations & Challenges

The high level of degradation as well as the heterogenous and unusual physical state of ancient samples pose a great challenge for the analysis of archaeological samples (Tanasi et al., 2021). The lack of chemical data on contemporary reference materials can hinder the identification of organic residue origins in archaeological samples. This warrants expanding investigations of the chemical composition of contemporary plant and animal reference materials to include those likely to have been significant in the past (Vykukal et al., 2024).

Nevertheless, a primary challenge in such investigations pertains to the degradation processes impacting biomolecules. The chemical and physical characteristics of the depositional environment can exert diverse influences on organic residues, in addition, contemporary contamination cannot be entirely discounted during chemical analysis, particularly in the case of non-sealed jars, even when employing the most sophisticated analytical instrumentation (D'Agostino et al., 2023).

The potential and nature of secondary metabolite degradation in plant residues, such as resins, gums, tars, essential oils, spices, herbs, and psychoactive plant products, have been relatively underexplored to this day (Huber et al., 2022).

The analysis of archaeological lipids presents several challenges, where the degradation of original lipids due to factors such as oxidation and hydration in the burial environment, as well as from past food processing, particularly cooking, poses identification challenges (Cramp et al., 2023). The lipid pattern therefore represents the accumulation of the pot's lifetime usage, which can result in extremely complicated lipid patterns. Furthermore, use-related alterations and post-depositional degradation complicate lipid patterns even further. As a result, lipid extracts from archeological artifacts can include several hundred different chemicals, making analysis and interpretation extremely complex (Korf et al., 2020).

In the context of heavily degraded ancient samples, ancient DNA (aDNA) research faces numerous challenges, including sample heterogeneity, limited DNA quantity, fragmentation, degradation, contamination, post-mortem damage, and chemical modifications. These factors collectively pose significant obstacles and introduce biases in downstream analyses, thereby complicating the integration of data (Boggi et al., 2023).

The provenance of the majority of archaeological artifacts over the millennia is challenging to ascertain, and contemporary contamination cannot be entirely discounted during chemical analysis, particularly in the case of non-sealed jars, even when employing the most sophisticated analytical instrumentation (Hammann et al., 2020). Further, the volatility of essential diagnostic plant compounds, viz. sesquiterpenoids can hinder the differentiation of plant species (Huber et al., 2022).

During sample collection and preparation, it is not possible to rule out the presence of endogenous compounds or modern contaminants (Hammann et al., 2020), including human-origin contamination, mainly due to improper handling of archaeological findings without gloves which might hinder data interpretation, especially DNA (Vilanova & Porcar, 2020).

In archaeological settings, adverse alteration of plant substances by organic or inorganic catalysts may occur when organic compounds react with the surfaces or materials of ancient containers and vessels, such as clay, gypsum, and various metals. These reactions can entail modifications in covalent bonds, such as condensations, the addition or removal of oxygenated groups, intramolecular migration or replacement of functional groups (substitution), desaturation reactions, or bond breakage (elimination). Biomarker analysis and data interpretation must take into account all of these processes and their ability to alter the molecular structure of a specific residue (Huber et al., 2022).

Proteomic techniques can only be applied to well-preserved archaeological materials from cold, dry, or wet environments (Tanasi et al., 2021).

## 9 Artificial intelligence & future directions

The field of ancient biomolecules is on the brink of exploring exciting new avenues in the coming years, promising significant advancements in our understanding and applications.

Investigations into the elemental and macromolecular composition have yielded positive results, indicating that the study of biomolecules continues to be a feasible approach in the field of archaeology (Badillo-Sanchez, Ruber, Davies-Barrett, Jones, & Inskip, 2023).

Algorithms for artificial intelligence can undergo training to identify and sort various artifacts using images. This capability can assist archaeologists in promptly categorizing and identifying objects unearthed at archaeological sites (Vodanović et al., 2023).

The integration of artificial intelligence within the field of archaeology presents a substantial opportunity to enhance our comprehension of historical contexts. This technological application facilitates the acquisition of novel insights into the lives and cultures of our ancestors. Further, biomolecular analyses also address the critical question of how context-informed scientific analysis can contribute to bridging existing gaps in archaeological research. Furthermore, this approach has the potential to reassess established historical models by challenging conventional understandings of the functions and contents of various containers (Polla & Springer, 2022; Vodanović et al., 2023).

The analysis and interpretation of extensive data from historical sites and artifacts could be revolutionized by artificial intelligence, potentially transforming the field of archaeology. Analyzing extensive genomic data using artificial intelligence allows bioarchaeologists and anthropologists to track the evolutionary history of various populations and pinpoint genetic markers linked to specific traits or conditions (Vodanović et al., 2023).

## 10 Conclusion

Archaeobotanical and archaeological remains serve as crucial windows into the past, providing rich insights into the social dynamics and environmental conditions that shaped ancient societies. These remnants—ranging from seeds and plant fibers to soil and pottery—offer invaluable evidence of what life was like, revealing details about agriculture and daily activities in bygone eras. Hence, investigating these artifacts allow for understanding human history and how ancient civilizations interacted with their surroundings. Archaeological excavations at the Helwan cemetery led by the Egyptologist Zaki Saad encompass a wide array of archaeological and archaeobotanical remains that date back to early dynasties. The interlinking between similar types of remains found in different sites around the world and their analytical findings might contribute to our understanding of ancient dietary habits that we can reflect on and apply to Zaki Saad collectibles. Biomolecular archaeology plays a crucial role in uncovering the mysteries of our past by analyzing ancient molecules, such as nucleic acids, proteins, lipids, stable isotopes, and carbohydrates. By studying them, we can unlock answers to long-standing questions about human history, providing invaluable insights into our origins and evolution. Recent advancements in multi-omics techniques—specifically genomics, metabolomics, proteomics, and lipidomics—have facilitated the generation of high-throughput biomolecular data in archaeology. These techniques, combined with the analysis of extensive data from historical sites and artifacts, could be enhanced by artificial intelligence, potentially transforming the field of archaeology. In this review, we provide a comprehensive overview of the state-of-the-art analytical platforms used in isotope analysis and multi-omics research within biomolecular archaeology. Additionally, we discuss the limitations and challenges faced by these analytical platforms.

## CRediT authorship contribution statement

**Nehal S. Ramadan:** Writing – review & editing, Writing – original draft, Visualization, Supervision, Project administration, Funding acquisition, Conceptualization. **Magdy M. El-Sayed:** Writing – review & editing, Writing – original draft, Visualization, Supervision, Project administration, Funding acquisition, Conceptualization. **Hesham Sameh Ramadan:** Writing – review & editing, Writing – original draft. **Mostafa Ismail:** Writing – original draft. **Heba Abdelmegeed:** Writing – review & editing, Writing – original draft. **Nashwa Gaber:** Project administration. **Mahmoud M. Sakr:** Writing – original draft, Visualization, Supervision, Project administration, Funding acquisition, Conceptualization.

## Informed consent statement

Not applicable.

## Institutional review board statement

Not applicable.

## Declaration of generative AI and AI-assisted technologies in the writing process

During the preparation of this work the authors used Gemini in order to improve language and readability. After using Gemini, the author(s) reviewed and edited the content as needed and take(s) full responsibility for the content of the publication.

Magdy M. El-Sayed reports financial support was provided by Academy of Scientific Research & Technology. If there are other authors, they declare that they have no known competing financial interests or personal relationships that could have appeared to influence the work reported in this paper.

## Funding

This research received funding from the Academy of Scientific Research and Technology (ASRT).

## Declaration of competing interest

The authors declare that they have no known competing financial interests or personal relationships that could have appeared to influence the work reported in this paper.

## Data Availability

No data was used for the research described in the article.
